# Determination of personal care products and hormones in leachate and groundwater from Polish MSW landfills by ultrasound-assisted emulsification microextraction and GC-MS

**DOI:** 10.1007/s11356-015-5359-9

**Published:** 2015-09-18

**Authors:** Justyna Kapelewska, Urszula Kotowska, Katarzyna Wiśniewska

**Affiliations:** Institute of Chemistry, University of Bialystok, ul. Ciołkowskiego 1K, 15-245 Bialystok, Poland

**Keywords:** Endocrine disrupting compounds, Hormones, Personal care products, Groundwater, Landfill leachate, Ultrasound-assisted emulsification microextraction, Gas chromatography–mass spectrometry

## Abstract

Determination of the endocrine disrupting compounds (EDCs) in leachate and groundwater samples from the landfill sites is very important because of the proven harmful effects of these compounds on human and animal organisms. A method combining ultrasound-assisted emulsification microextraction (USAEME) and gas chromatography–mass spectrometry (GC-MS) was developed for simultaneous determination of seven personal care products (PCPs): methylparaben (MP), ethylparaben (EP), propylparaben (PP), buthylparaben (BP), benzophenone (BPh), 3-(4-methylbenzylidene)camphor (4-MBC), *N*,*N*-diethyltoluamide (DEET), and two hormones: estrone (E1) and β-estradiol (E2) in landfill leachate and groundwater samples. The limit of detection (LOD)/limit of quantification (LOQ) values in landfill leachate and groundwater samples were in the range of 0.003–0.083/0.009–0.277 μg L^−1^ and 0.001–0.015/0.002–0.049 μg L^−1^, respectively. Quantitative recoveries and satisfactory precision were obtained. All studied compounds were found in the landfill leachates from Polish municipal solid waste (MSW) landfills; the concentrations were between 0.66 and 202.42 μg L^−1^. The concentration of pollutants in groundwater samples was generally below 0.1 μg L^−1^.

## Introduction

Along with the civilization development and the evolution of human lifestyle, there was an increase in consumption and thus the formation of more and more wastes of diversified composition. Despite the emerging research on the risks associated with the solid waste landfills, there are still plenty of unresolved issues concerning the negative effects of their operation. This is mainly due to changes in the solid waste (Renou et al. 2008). The landfill leachate is a heterogeneous matrix, formed by excess water percolating through the waste layers in the landfills, and is commonly referred to as “difficult wastewater” (Foo et al. 2013). The ecotoxicological studies of landfill leachates demonstrate their acute toxicity (Alkassasbe et al. 2009; Kalka 2012). In municipal solid waste landfills, various kinds of material are disposed of. They include waste products containing endocrine disrupting compounds (EDCs) and incineration residue that includes dioxin (Asakura et al. 2004). In the case of an insufficient insulation system between the mass of landfilled waste and the soil environment, organic pollutants can easily seep into ground and surface water, and to drinking water (Nomngongo et al. 2012; Rosi-Marshall and Royer 2012). This is one of the most important issues associated with the impact of the landfill on the aquatic environment. This threat is all the more real, because many landfill sites in Poland use the so-called natural insulating barrier, which does not adequately protect the aquatic environment from organic pollutants.

Standard environmental monitoring measures applied toward landfill leachate or seepage mainly include the determination of bulk parameters, such as total organic carbon, dissolved organic carbon, chemical oxygen demand, biological oxygen demand as well as element analysis of anions, cations, and heavy metals (Preiss et al. 2012). The problems of the occurrence of EDCs in the landfill leachate from municipal solid waste (MSW) landfill were undertaken by scientists only several times so far. This may be connected to the fact that the detection of trace compounds in landfill leachate is a difficult task, due to the very complex matrix of leachate, more complicated than majority of liquid environmental samples. Landfill leachate contains large amounts of inorganic salts, heavy metals, nitrogen compounds, and different kinds of organic compounds, including humic substances, which cause their dark color. In previous publications, designations of phthalates, octylphenol, nonylphenol, bisphenol A, and polybrominated diphenyl ethers (PBDEs) in landfill leachate have been described (Yamamoto et al. 2001; Asakura et al. 2004; Dos Santos et al. 2004; Kurata et al. 2008; Odusanya et al. 2009; Kwan et al. 2012; Zhang et al. 2012; Kalmykova et al. 2013). Concentrations of target EDCs in the landfill leachate fit within a very wide range from a few nanograms per liter up to tens of milligrams per liter.

Methylparaben (MP), ethylparaben (EP), propylparaben (PP), and buthylparaben (BP) are used as preservatives in pharmaceutical, personal care, and food products. In the European Union (EU), the use of parabens in cosmetics is limited to a maximum concentration of 0.4 % (*w*/*w*) for one type of parabens and of 0.8 % (*w*/*w*), expressed as p-hydroxybenzoic acid, for parabens mixtures (European Commission 1976). Regarding their toxicological effects, these compounds have shown estrogenic activity and are potentially toxic for certain aquatic organism; however, it is relatively weak in comparison to toxicity of E2 (Harvey and Everett 2004). Benzophenone (BPh) and 3-(4-methylbenzylidene)camphor (4-MBC) are sunscreen agents. They have the ability to absorb and dissipate ultraviolet light. Therefore, it is used in many cosmetics and sunscreens to protect human skin from ultraviolet radiation (Pietrogrande and Basaglia 2007). Diethyltoluamide (DEET) is one of the most widespread and efficient insect repellents (e.g., mosquito). DEET is known to be persistent (Costanzo et al. 2007) but weakly toxic toward fishes, birds, and invertebrates (Pietrogrande and Basaglia 2007). E1 and E2 are examples of natural estrogen found in birth control pills. Prenatal exposure to both natural and synthetic estrogens has been associated with increased occurrence of vaginal and breast tumors in humans and uterine tumors in animals. Exposure to natural steroid hormones will likely elicit an effect because these hormones can readily bind to receptors to activate transcription and protein synthesis (Streets 2008).

MP, EP, PP, BP, and BPh were found in different environmental samples: tap water, surface water, and influent and effluent wastewater (Trenholm et al. 2008; Regueiro et al. 2009b; Casas Ferreira et al. 2010; Gracia-Lor et al. 2012; Kotowska et al. 2013; Zhang and Lee 2013). DEET was found in river water, sea water, and wastewater samples (Trenholm et al. 2008; Calza et al. 2011; Kotowska et al. 2013; Loos et al. 2013). E1 and E2 were detected in river water and influent and effluent wastewater samples (Kumar et al. 2009; Kotowska et al. 2013). 4-MBC was found in tap water and influent and effluent wastewater so far (Liu et al. 2011, 2012; Kotowska et al. 2013). There is no information on the presence of hormones and personal care products (PCPs) in the landfill leachate from MSW landfill. Furthermore, there is no information about the determination of EDCs in groundwater samples collected from piezometers located under the landfill sites.

Determination of the hormones and PCPs in environmental samples is a very difficult task due to their low concentrations and complex matrix of the substances. In most of cases, these contaminants are present at levels of several nanograms per liter to several micrograms per liter (Trenholm et al. 2008; Calza et al. 2011; Gracia-Lor et al. 2012; Liu et al. 2012). Only in the last decades has been developed the analytical methods for the determination of compounds present in the environment in ultratrace quantities. The most extensively used technique for the isolation of the EDCs from the environmental samples is the solid-phase extraction (SPE) (Trenholm et al. 2008; Calza et al. 2011; Gracia-Lor et al. 2012; Liu et al. 2012; Loos et al. 2013). However, application of SPE is time-consuming and labor-intensive and needs a quite large amount of organic solvents, specially comparing with microextraction techniques. This is why more and more frequently, there is a need for methods enabling to eliminate or substantially reduce the consumption of organic solvents during the analytical procedure. To isolate BPh and 4-MBC, apart from SPE, also dispersive liquid-phase microextraction (DLPME) and vortex-assisted liquid–liquid microextraction (VALLME) are applied (Zhang and Lee 2012, 2013). In the case of parabens, also other techniques have been applied, such as solid-phase microextraction (SPME) (Regueiro et al. 2009a), stir bar sorptive extraction (SBSE) (Casas Ferreira et al. 2010), and ultrasound-assisted emulsification microextraction (USAEME) (Regueiro et al. 2009b). The USAEME technique is variant liquid–liquid microextraction (LLME) and was first used by Regueiro 7 years ago (Regueiro et al. 2008). USAEME techniques are now widely employed due to a great number of advantages that they offer, such as low consumption of organic solvents, simplicity of experiment, high extraction efficiency, and low costs. The final stage of the determination of EDCs in environmental samples requires extremely sensitive and selective techniques. Currently, for this purpose, chromatographic methods are primarily used, i.e., gas chromatography (GC), high-performance liquid chromatography (HPLC), and ultrahigh-performance liquid chromatography (UHPLC). The detector typically employed in conjunction with chromatographic techniques is mass spectrometer (MS). Another detector applied and used in liquid chromatographic assays is ultraviolet detector (UV).

The aim of this work was to develop a quick and selective analytical method to carry out the determination of seven compounds from the group of PCPs (i.e., MP, EP, PP, BP, BP, DEET, and 4-MBC) and two hormones (i.e., E1 and E2) at low concentrations. USAEME has been used for the isolation whereas GC-MS in the selected ion monitoring (SIM) mode has been applied for the separation and determination of analytes. The effect of various extraction and derivatization parameters, i.e., the type and volume of organic solvent, extraction time, derivatization reagent volume, and kind and amount of buffering salt was investigated. The developed USAEME/GC-MS method was employed to determine target compounds in landfill leachate and groundwater samples from MSW landfill sites in Poland.

## Materials and methods

### Reagents and solvents

MP, EP, PP, BP, BPh, 4-MBC, DEET, E1, E2, octanol, decane, and urea were obtained from Sigma-Aldrich (Germany). Carbon tetrachloride was purchased from Merck (Germany). Chloroform, toluene, cyclohexane, sodium hydrogen phosphate (V), magnesium sulfate (VI) anhydrous, sodium bicarbonate, anhydrous potassium carbonate, sodium nitrate (V), and methanol were obtained from POCH (Poland). Acetic anhydride, ammonium bicarbonate (IV), magnesium chloride, potassium hydrogen phosphate (V), potassium carbonate, and calcium chloride anhydrous were obtained from Chempur (Poland). Stock solutions of each analyte (at 1 mg mL^−1^ each) were prepared separately in methanol and stored at −18 °C not longer than 1 month. Working solutions were prepared by diluting the stock standard solution in methanol and stored at −18 °C not longer than 2 weeks. Deionized water was from purification system (Milli-Q RG, Millipore, USA) and was stored in glass bottle.

### Synthetic landfill leachate

The synthetic landfill leachate was prepared in order to eliminate effects of the target compounds present in the real landfill leachate on the sensitivity of the method. The recipe was adapted from the research conducted by Champagne and Li (2009). The synthetic landfill leachate was prepared by dissolving the corresponding analytical grades of chemicals in distilled water as shown in Table 1.

**Table 1:** Synthetic landfill leachate composition

Constituents	mg L^−1^
K_2_HPO_4_	30
KHCO_3_	31
K_2_CO_3_	324
NaNO_3_	50
NaHCO_3_	3012
CaCl_2_	2176
MgCl_2_·6 H_2_O	3114
MgSO_4_	156
NH_4_HCO_3_	2439
CO(NH_2_)_2_	695
CH_3_COOH	7350
NaOH	to a pH ≈ 9.60

### Real samples

The landfill leachate was obtained from the three MSW landfill sites, and groundwater samples were collected from the two MSW landfill sites, all located in northeastern Poland. The samples were obtained from landfills for non-hazardous and inert waste, with different characteristics (different in size, kind of insulation, the method of collecting leachate, and age). Table 2 shows characteristics of studied MSW landfills. In MSW landfill site A, the leachate is stored in opened lagoons, while in landfills B and C, leachate is stored in underground wells. The examined landfills discharged leachate to a wastewater treatment plant by tanker trucks. Additionally, in landfill B, part of the leachates is recycled by spraying on the landfill cap. The groundwater samples were collected from piezometers located under the landfill sites. Samples of landfill leachate and groundwater samples were collected between April 2012 and May 2013. Samples were introduced into glass bottles and transported to the laboratory. All bottles and equipment used to collect leachate and groundwater samples were cleaned using an anionic detergent and were thoroughly rinsed with tap water followed by deionized water. After that, sampling bottles and equipment were rinsed with pesticide-free methanol and allowed to air dry. Upon arrival, the samples were filtered through a membrane filter with 0.45 μm pore size and acidified with concentrated hydrochloric acid to pH = 2. Later, the real samples were stored at −18 °C.

**Table 2:** Characteristics of study MSW landfills (Inspection of Environmental Protection and Inspectorate of Environmental Protection in Bialystok 2013)

	Status	Capacity (m^3^)	Kind of insulation	Number of piezometers	Class^a^ of groundwater in 2011	Class^a^ of groundwater in 2012
MSW landfill A	Open	242.311	Mixed insulation (artificial/natural)	4	V, V, V, V	V, V, V, I
MSW landfill B	Open	480.000	PVC foil	4	II, II	II, I, II, II
MSW landfill C	For the closure	45.092	n.d.	4	IV, V, V, IV	V, V, V, V

^a^Based on the Regulation of the Minister of Environment of 23 July 2008, on the criteria and methods of evaluation of groundwater samples (Dz.U. Nr 143 poz. 896)

*n.d.* no data

### The procedure of ultrasound-assisted emulsification microextraction with in situ derivatization

For the simultaneous USAEME and derivatization, aliquots of 5 mL water samples were placed in 10-mL glass centrifuge tubes containing previously weighted 0.1 g of sodium hydrogen phosphate. The extraction solvent (chloroform, 70 μL) and the derivatization reagent (acetic anhydride, 50 μL) were added to the water sample and mixed. Immediately after, the tube was immersed in an ultrasonic Sonic-3, Polsonic (Poland) water bath in such a way that the levels of both liquids (i.e., the bath and the sample) were equal. Extractions were performed at 42 kHz of ultrasound frequency and 230 W of power for the duration of 5 min at a room temperature. Emulsions were disrupted by centrifugation at 4000 rpm for 5 min in an MPW-250 Med. Instruments (Poland) laboratory centrifuge. In effect, the organic phase settled at the bottom of the conical tube. After centrifugation, chloroform was removed using a 100 μL Hamilton syringe (USA) and transferred into a 150 μL microvial with integrated insert.

To ensure the quality of results, the USAEME procedure was done in triplicate for all real samples as well as spiked samples used for method validation. Quality control samples used for method validation (synthetic landfill leachate and real groundwater samples) were examined for the presence of target compounds. Field blanks were prepared in field by processing deionized water through the sampling equipment in the same manner that real samples were collected. Three laboratory blanks and three field blanks were analyzed, and no one of nine target chemicals was detected.

### Gas chromatography–mass chromatography analysis

Analysis was performed with a HP 6890 gas chromatograph with a mass spectrometric detector MSD5973 and HP 7673 autosampler (Agilent Technologies, USA). This device was equipped with ZB-5MSi (5 % phenylmethylsiloxane) size 30 m length × 0.25 mm, i.e., coated with 0.25-μm film thickness and split/splitless injector. The injector worked in splitless mode. Helium of purity 99.999 % was used as carrier gas at flow rate 1 mL min^−1^. The injector temperature was 250 °C. The oven temperature was programmed from 150 °C, increased at 5 °C min^−1^ to 185 °C and 20 °C min^−1^ to 270 °C. The total run time was 17.25 min. The MS detector worked in SIM mode. The electron impact source temperature was 230 °C with electron energy of 70 eV. The quadrupole temperature was 150 °C, and the GC interface temperature was 280 °C. The retention times and molecular weights of target compounds are shown in Table 3 together with the quantification and identification ions.

**Table 3:** The retention times (*t*_R_), quantification and identification ions (*m*/*z*), and molecular weights (MWs) of analytes not undergoing acetylation and acetylated derivatives of analytes

Analyte	Retention time (*t*_R_) [min]	Quantification and identification ions (*m*/*z*)	Molecular weight (MW)
Analytes not undergoing acetylation			
DEET	5.85	91, **119**, 190	191
BPh	6.64	77, **105**, 182	182
4-MBC	10.76	128, 171, **254**	254
Acetylated derivatives of analytes			
MP	4.60	43, **121**, 152	194
EP	5.47	**121**, 138, 166	208
PP	6.86	121, **138**, 180	222
BP	8.21	121, **138**, 194	236
E1	16.16	185, **270**, 272	312
E2	16.42	43, 146, **272**	314

Bold ions selected for monitoring

## Results and discussion

### Optimization of extraction and derivatization procedure

All tests during the optimization process had been carried out using distilled water containing 100 μg L^−1^ of each target compounds.

#### Organic solvent selection

**Fig. 1:**
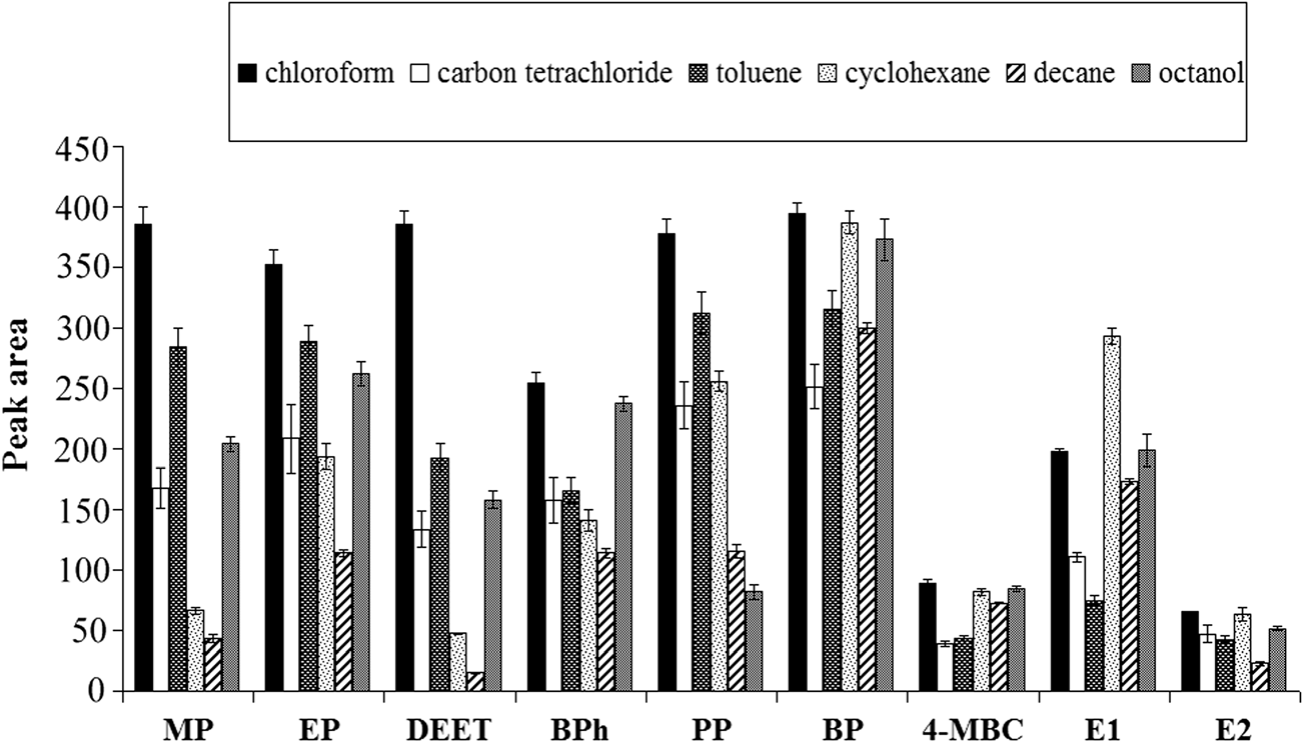
The influence of organic solvent on extraction efficiency of target compounds (*n* = 3)

Physicochemical properties of organic solvent, such as solubility in water, viscosity, and extraction capacity, have crucial impact on the efficiency of extraction process of target compounds. In the USAEME technique, the choice of solvent is also dictated by the possibility of forming emulsion during the extraction procedure. For application of the above conditions, chloroform, carbon tetrachloride, toluene, cyclohexane, *n*-decane, and 1-octanol were examined in the preliminary experiments. In their course, 70 μL of solvent was added to 5 mL aliquots of the target compound solution, and the samples were placed in an ultrasonic bath for 5 min. Emulsification was observed in all of the cases. After centrifugation, the extraction solvents with density higher than water (carbon tetrachloride and chloroform) were removed with syringe from the conical bottom of the test tube. Toluene, cyclohexane, *n*-decane, and 1-octanol, which have lower density than water, were collected from the bottom of the tube after removal of water. The results of organic solvent selection are shown in Fig. 1. In cases of MP, EP, PP, BP, DEET, BPh, 4-MBC, and E2, the largest peak areas were obtained using chloroform, whereas in case of E1, the largest peak areas were obtained using cyclohexane as the extraction solvent. That is why chloroform was eventually selected as the optimum extraction solvent for further experiment.

#### Volume of extraction solvent

**Fig. 2:**
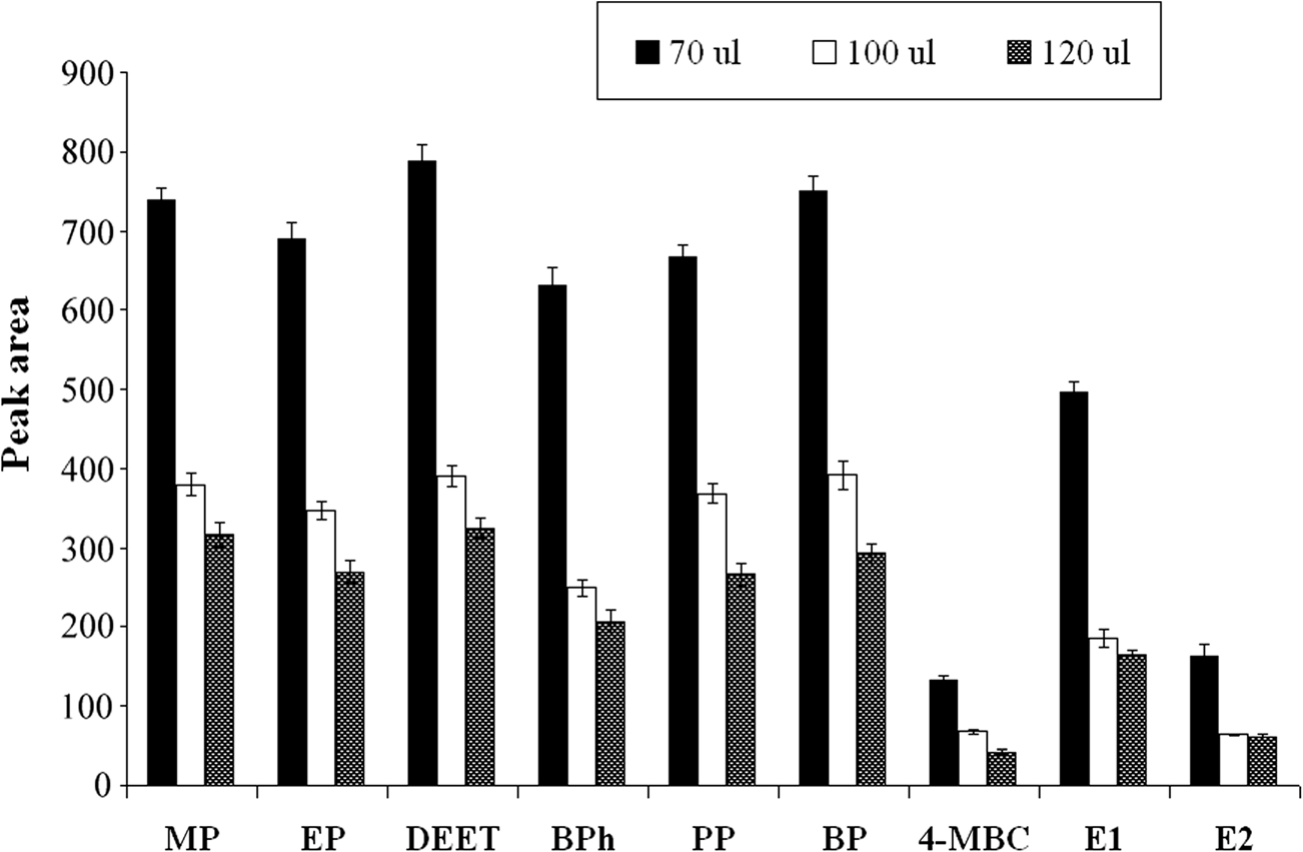
The influence of volume of extraction solvent on extraction efficiency of target compounds (*n* = 3)

The volume of extraction solvent is a crucial parameter that has an important effect on the extraction efficiency: The lower the volume of organic phase, the greater the concentration of the analyte, which effects in lowering the limit of quantification (LOQ). On the other hand, the amount of solvent recovered after the extraction process should be sufficient to carry out injection of the sample into the GC-MS device with the use of autosampler. To study the effect of extraction solvents, different volumes of chloroform (70, 100, and 120 μL) were subjected to the USAEME procedure (Fig. 2). The analysis showed that reduction of solvent volume is accompanied by growth of peak areas of the analyzed compounds. The amount of solvent recovered after the extraction process, conducted with the use of 70 μL of chloroform, ranged between 20 and 30 μL, and the used volume was the smallest which allowed introduction of the sample into chromatograph with autosampler. All in all, 70 μL chloroform as extractant solvent was used in subsequent experiments.

#### Effect of the extraction and derivatization time

**Fig. 3:**
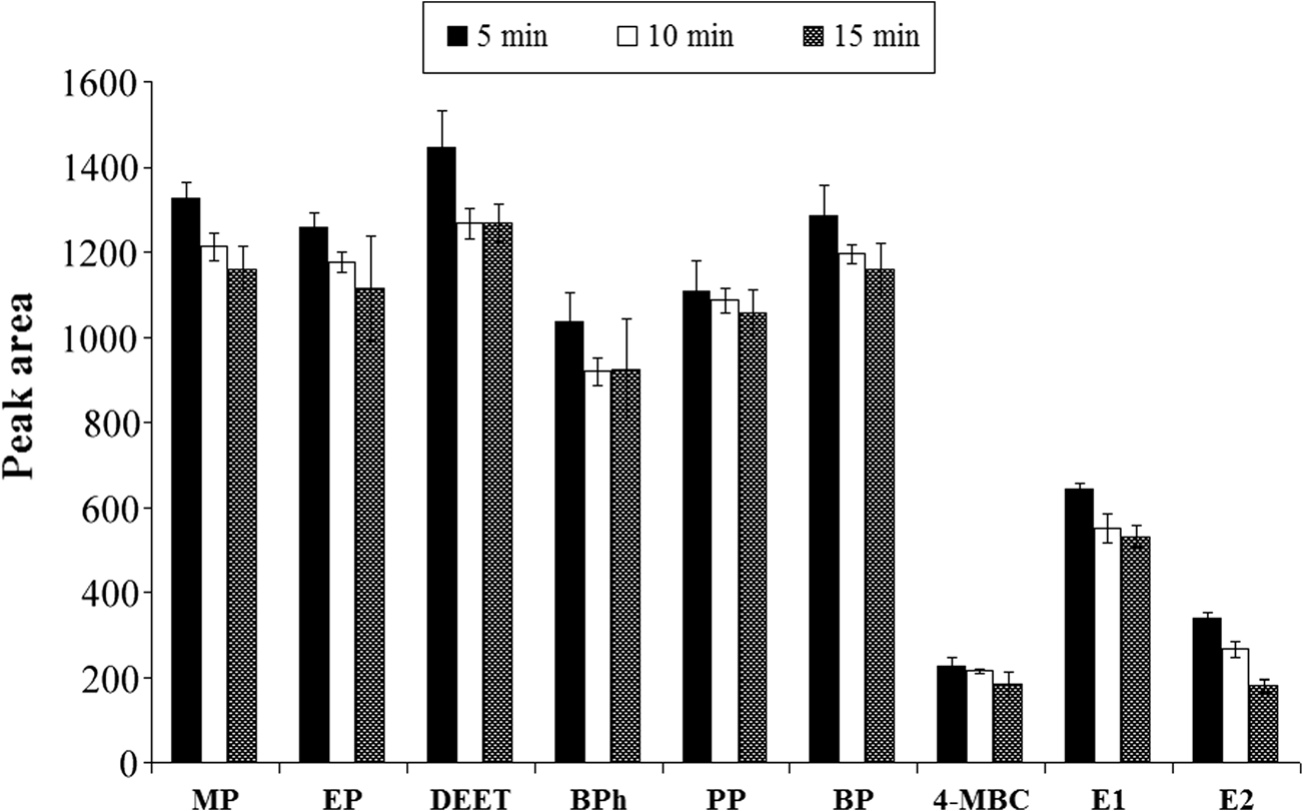
The influence of extraction times on extraction efficiency of target compounds (*n* = 3)

In USAEME, extraction time is defined as the time between injection of extraction solvent and the end of the sonication stage (Ma et al. 2009). The sonication time should provide the maximum dispersion which affects the extraction efficiency of the analyte. In the used procedure, at the same time, extraction and derivatization of analytes in the matrix were performed. An ultrasound-assisted process was adopted in a range of 5–15 min to evaluate its effect on extraction and derivatization (Fig. 3). Based on the obtained results, we found that there were no significant differences in signal intensities registered after individual extraction time, so the shortest extraction time 5 min was chosen as the most suitable for further studies. At this time, total derivatization of analytes occurred, which was confirmed by registered chromatograms and mass spectra.

#### Effect of kind and amount of buffering salt

**Fig. 4:**
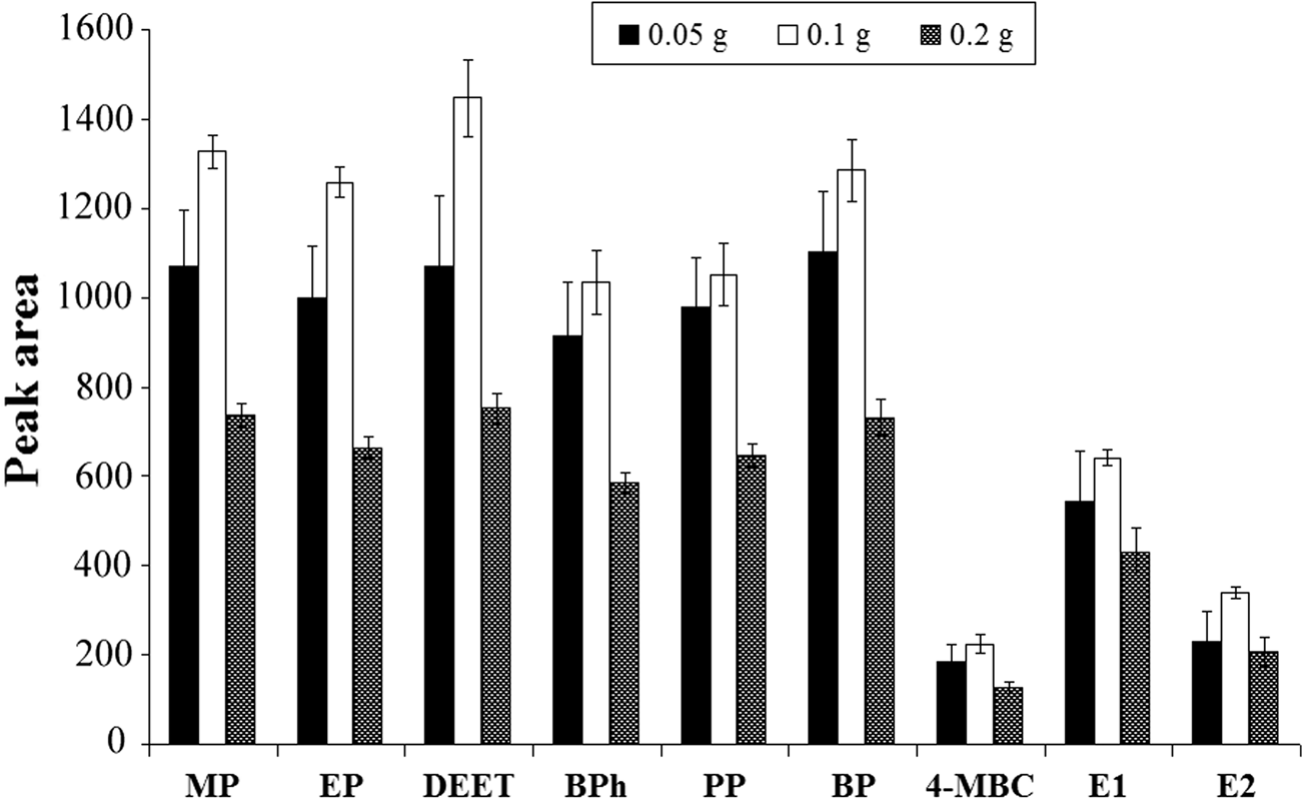
The influence of kind and amount of buffering salt on extraction efficiency of target compounds (*n* = 3)

In the derivatization technique with the use of acetic anhydride, an addition of buffer salt is necessary. Sodium hydrogen carbonate is the most frequently used for this purpose. However, in USAEME procedure, carbon dioxide bubbles are produced as a consequence of decomposition of carbonates interfering with the collection of the organic phase, so in the present work, sodium hydrogen phosphate was used as the buffering salt. To study the effects of various amounts of added sodium hydrogen phosphate on the derivatization process, experiments were performed with different quantities of this salt (0.05–0.2 g) per 5 mL of wastewater sample. Figure 4 illustrates the influence of added quantity of sodium hydrogen phosphate on extraction efficiency of the target compounds. It can be noticed that the highest peak areas were obtained using 0.1 g of sodium hydrogen phosphate; therefore, 0.1 g was used in subsequent experiments.

#### Effect of derivatization reagent volume

**Fig. 5:**
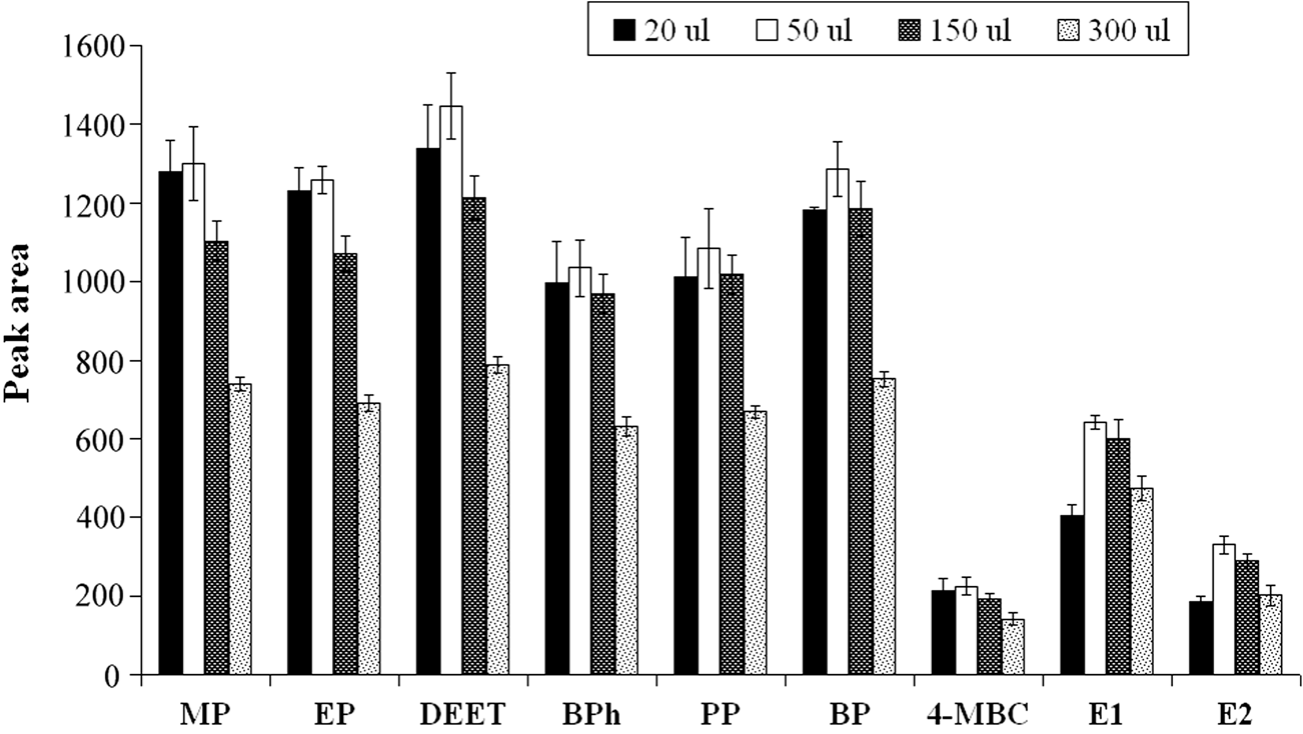
The influence of derivatization reagent volume on extraction efficiency of target compounds (*n* = 3)

The influence of the volume of acetic anhydride on the relative peak area was studied in the range of 20–300 μL (Fig. 5). The results indicated that the volume of acetic anhydride equal to 50 μL should be chosen as optimal.

### Method validation

To evaluate the developed USAEME-GC/MS method, the linearity, LOQ, and limit of detection (LOD), repeatability and recovery were investigated under the optimum conditions. The validation method was conducted by using deionized water as the sample matrix (Table 4) and also groundwater and synthetic landfill leachate as the sample matrix (Table 5). The calibration curves were built by spiking the synthetic landfill leachate and groundwater samples with 5 to 11 levels of concentration (depending on the analyzed compound and the kind of matrix used) and performing the extraction. The calibration curves were built on two levels of concentration for MP, EP, PP, BP, BPh, and DEET and on one level of concentration for 4-MBC, E1, and E2. Calibration curves were linear within the studied concentration range, with coefficients of correlation higher than 0.981 for all the target compounds. The LOQ, determined as the analyte concentration corresponding to a signal/noise ratio of 10, were between 0.002 and 0.049 μg L^−1^ by using groundwater as sample matrix and were between 0.001 and 0.278 μg L^−1^ by using synthetic landfill leachate as sample matrix. The LOD, defined as the concentration that corresponds to three times standard deviation of blanks, were between 0.001 and 0.015 μg L^−1^ by using groundwater as sample matrix and were between 0.003 and 0.083 μg L^−1^ using synthetic landfill leachate as sample matrix. Relative standard deviation (RSD) and recoveries were tested for two concentration levels (0.09 and 8 μg L^−1^ in cases of groundwater samples and 0.8 and 7 μg L^−1^ in cases of synthetic landfill leachate samples). These values are presented in Table 5. RSD were below 21 % for the lower concentration level, and RSD were below 16 % for the higher concentration level. Recoveries were evaluated by spiking matrix before and after extraction; then, the obtained signals were compared. In the case when groundwater was used as the sample matrix, recoveries were between 85 and 136 %. When using landfill leachate as the sample matrix, the recoveries were between 83 and 136 %.

**Table 4:** Analytical characteristics of the USAEME/GC-MS method

Analyte	Linearity (μg L^−1^)				LOD (μg L^−1^)	LOQ (μg L^−1^)	RSD (%, *n* = 3)		Recovery (%, *n* = 3)	
	Range I	*R* ^2^	Range II	*R* ^2^			0.02 μg L^−1^	7 μg L^−1^	0.02 μg L^−1^	7 μg L^−1^
MP	0.005–0.05	0.9896	0.05–10	0.9975	0.0006	0.0020	21.5	11.4	115.5	100.8
EP	0.005–0.05	0.9811	0.05–10	0.9967	0.0011	0.0038	20.9	8.9	78.7	98.4
PP	0.005–0.05	0.9749	0.05–10	0.9984	0.0004	0.0013	24.4	12.0	89.0	102.1
BP	0.001–0.05	0.9963	0.05–10	0.9971	0.0001	0.0004	23.0	11.2	116.2	104.0
BPh	0.001–0.05	0.9938	0.05–10	0.9972	0.0003	0.0008	24.2	11.6	115.1	106.1
4-MBC	0.005–0.05	0.9782	0.05–10	0.9931	0.0015	0.0048	17.4	7.8	72.5	100.4
E1	0.005–0.05	0.9926	0.05–10	0.9975	0.0014	0.0048	23.6	9.7	103.0	99.7
E2	0.005–0.05	0.9998	0.05–10	0.9931	0.0014	0.0046	23.1	10.2	124.2	103.8
DEET	0.005–0.05	0.9861	0.05–10	0.9913	0.0006	0.0019	23.6	13.2	127.4	103.0

*LOD* limit of detection, *LOQ* limit of quantification

**Table 5:** Analytical characteristics of the USAEME/GC-MS method obtained with the use of groundwater and synthetic landfill leachates as the sample matrix

Analyte	Linearity (μg L^−1^)				LOD (μg L^−1^)	LOQ (μg L^−1^)	RSD (%, *n* = 3)		Recovery (%, *n* = 3)	
	Range I	*R* ^2^	Range II	*R* ^2^						
Groundwater							0.09 μg L^−1^	8 μg L^−1^	0.09 μg L^−1^	8 μg L^−1^
MP	0.005–0.05	0.9910	0.05–10	0.9915	0.001	0.002	15	11	109	98
EP	0.005–0.05	0.9921	0.05–10	0.9922	0.001	0.004	21	9	119	91
PP	0.005–0.05	0.9931	0.05–10	0.9902	0.001	0.003	18	13	96	105
BP	0.005–0.05	0.9996	0.05–10	0.9950	0.001	0.003	20	13	93	108
BPh	0.005–0.05	0.9875	0.05–10	0.9946	0.001	0.002	18	11	113	107
4-MBC	0.05–10	0.9806	–	–	0.014	0.047	14	10	85	103
E1	0.05–10	0.9976	–	–	0.005	0.017	16	12	136	103
E2	0.05–10	0.9933	–	–	0.015	0.049	16	12	124	90
DEET	0.005–0.05	0.9925	0.05–10	0.9905	0.001	0.003	20	14	114	108
Synthetic landfill leachate							0.8 μg L^−1^	7 μg L^−1^	0.8 μg L^−1^	7 μg L^−1^
MP	0.05–2	0.9924	2–12	0.9967	0.010	0.033	13	12	96	104
EP	0.05–2	0.9976	2–12	0.9959	0.016	0.053	17	11	83	90
PP	0.03–2	0.9933	2–12	0.9973	0.009	0.030	18	15	110	96
BP	0.05–2	0.9963	2–12	0.9969	0.014	0.046	16	1	115	110
BPh	0.03–2	0.9991	2–12	0.9988	0.001	0.013	13	12	111	109
4-MBC	0.5–12	0.9941	–	–	0.056	0.188	14	13	88	109
E1	0.5–12	0.9944	–	–	0.036	0.121	15	13	127	91
E2	0.5–12	0.9970	–	–	0.083	0.278	16	15	136	83
DEET	0.01–1	0.9899	1–12	0.9953	0.003	0.009	20	16	118	109

*LOD* limit of detection, *LOQ* limit of quantification

### Environmental sample analysis

**Fig. 6:**
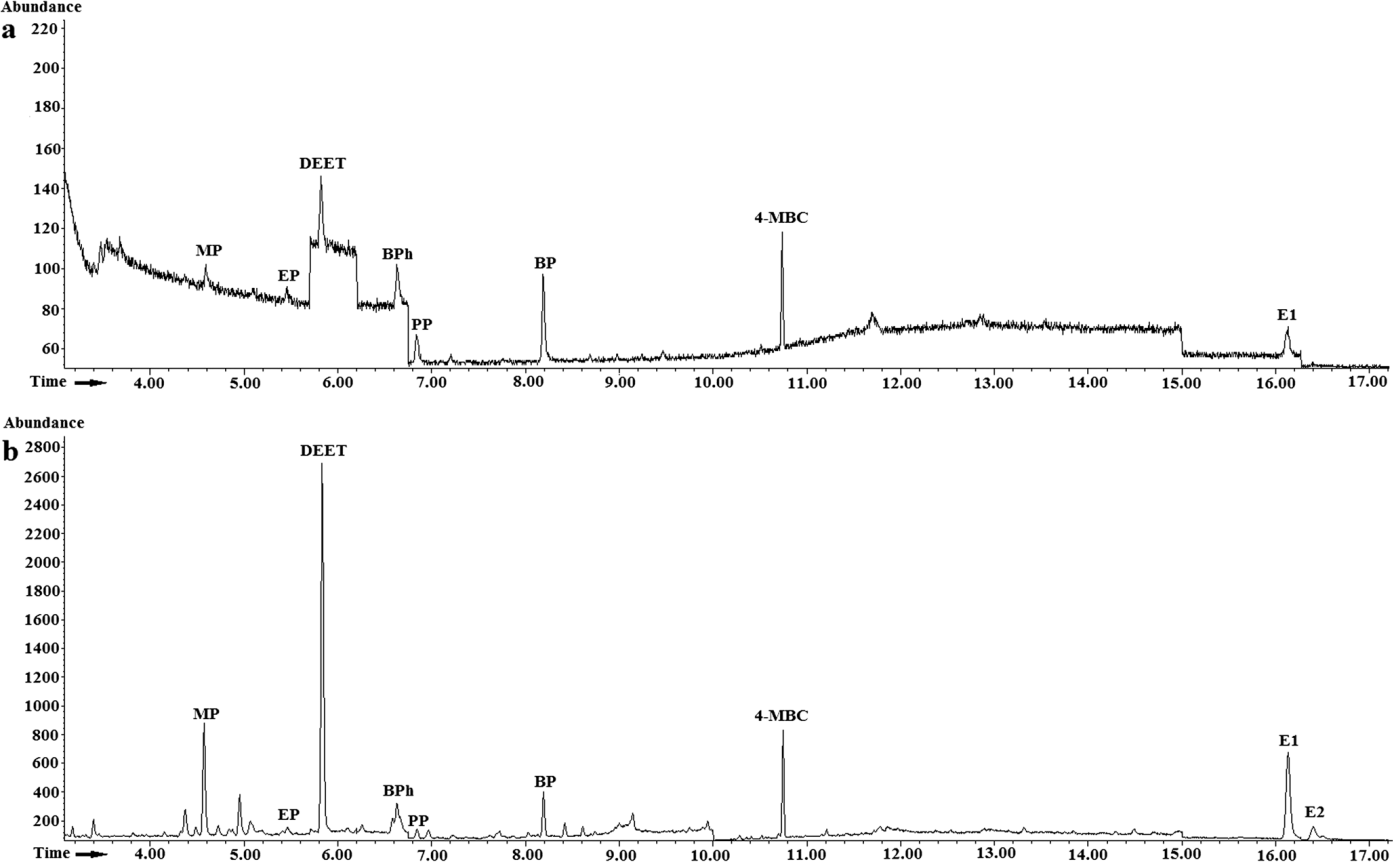
The chromatograms registered during analysis of groundwater (**a**) and landfill leachate (**b**) samples from MSW landfill B

The developed USAEME/GC-MS method was applied for determination of MP, EP, PP, BP, BPh, 4-MBC, DEET, E1, and E2 in landfill leachate obtained from three MSW landfills and groundwater samples from two MSW landfill sites in northeast Poland. The chromatograms of landfill leachate (a) and groundwater samples (b) from MSW landfill B are shown in Fig. 6. The occurrence of target compounds and levels of contamination in the analyzed water samples are summarized in Table 6. The concentrations of the target compounds in landfill leachate were determined from calibration curves registered by analysis of spiked synthetic landfill leachate samples and the concentrations of the target compounds in groundwater. They were calculated on the basis of the calibration curve registered by the use of groundwater samples.

**Table 6:** Concentration of the target endocrine disrupting compounds in landfill leachates and groundwater samples from MSW landfill located in northeastern Poland

Analyte	Range of concentration (μg L^−1^; *n* = 3)						
	Landfill leachate				Groundwater		
	MSW landfill A (NS = 5)	MSW landfill B (NS = 2)	MSW landfill C (NS = 2)	Median in leachate	MSW landfill A (NS = 12)	MSW landfill B (NS = 4)	Median in groundwater
MP	1.15–3.86	3.33–17.15	1.07–1.59	2.35	0.036–0.459	<LOQ–0.305	0.060
EP	2.24–9.38	1.10–5.19	0.66–1.19	2.24	<LOD–0.086	<LOD–0.064	0.070
PP	1.65–5.90	0.69–1.77	0.93–2.31	1.91	0.004–0.012	0.003–0.025	0.010
BP	1.22–6.67	0.75–2.30	0.80–2.38	1.55	<LOD–0.019	<LOD–0.032	0.012
BPh	0.72–3.63	0.95–3.33	1.13–3.86	1.98	0.028–0.492	0.038–0.068	0.062
4-MBC	1.22–16.64	2.81–6.18	5.20–7.79	3.77	<LOD–3.625	<LOD–2.383	0.262
E1	<LOD–0.17	<LOD–0.12	<LOD–0.12	0.12	<LOD–0.026	<LOD–0.043	0.026
E2	<LOD–0.28	<LOD	<LOD	0.24	<LOD–0.048	<LOD	0.039
DEET	11.41–132.97	28.27–202.42	32.92–101.71	32.83	0.019–16.901	0.023–0.047	0.053

*NS* number of samples, *LOQ* limit of quantification, *LOD* limit of detection

The PCP compounds were found in analyzed landfill leachate samples with 100 % frequency. The concentrations were between 0.66 and 17.67 μg L^−1^ (median between 1.55 and 3.77 μg L^−1^), except DEET which was detected in leachates from all the examined landfills in concentrations reaching hundreds of micrograms per liter (median 32.83). The average values of the concentration of target compounds were found on the level of several micrograms per liter, except DEET with the average value 71.16 μg L^−1^. Hormones were detected in landfill leachate samples in concentration reaching hundreds of nanograms per liter (median 0.122 μg L^−1^ for E1 and 0.238 μg L^−1^ for E2). E1 was detected and quantified in one sample from each landfill (frequency 33 %), while E2 was detected in two leachate samples from landfill A (frequency 22 %). The concentrations of determined PCP compounds in groundwater samples varied from values below LOD to few micrograms per liter except DEET and 4-MBC in which concentrations were larger than 1 μg L^−1^ in the same samples (median between 0.01 and 0.262 μg L^−1^). PP, BPh, and DEET were detected in 100 % of groundwater samples, while MP and 4-MBC were quantified in 63 %; EP and BP were quantified in 25 % of groundwater samples. Hormones were detected in groundwater samples in concentrations reaching several dozen of nanograms per liter. E1 was quantified in 19 % of groundwater samples—two samples from landfill A and one sample from landfill B. E2 was detected in two groundwater samples from landfill A (frequency 12 %).

## Conclusion

In the present study, an analytical methodology based on ultrasound-assisted emulsification microextraction followed by GC-MS determination has been proposed for determination of natural hormones, parabens, BPh, DEET, and 4-MBC. In situ derivatization with acetic anhydride conducted simultaneously with extraction was successful under the optimized conditions. The proposed USAEME technique with in situ derivatization step offers several advantages in terms of simplicity, low cost, minimal solvent consumption, and very short time of sample preparation. The proposed method was successfully applied for determination of the studied PCPs and hormones in landfill leachate and groundwater samples obtained from three municipal solid waste landfill sites in northeast Poland. All the studied compounds were found in the leachates from Polish MSW landfills; the concentrations were between hundredths of micrograms per liter and several hundreds of micrograms per liter. The concentrations of pollutants in groundwater samples varied from values below LOD to several of micrograms per liter.

## References

[CR1] Alkassasbe JYM, Heng LY, Surif S (2009). Toxicity testing and the effect of landfill leachate in Malaysia on behavior of common carp (Cyprinus carpio L., 1758; Pisces, Cyprinidae). Am J Environ Sci.

[CR2] Asakura H, Matsuto T, Tanaka N (2004). Behavior of endocrine-disrupting chemicals in leachate from MSW landfill sites in Japan. Waste Manage.

[CR3] Calza P, Medana C, Raso E, Giancotti V, Minero C (2011). N,N-diethyl-m-toluamide transformation in river water. Sci Total Environ.

[CR4] Casas Ferreira AM, Möder M, Fernández Laespada ME (2010). GC-MS determination of parabens, triclosan and methyl triclosan in water by in situ derivatisation and stir-bar sorptive extraction. Anal Bioanal Chem.

[CR5] Champagne P, Li C (2009). Use of Sphagnum peat moss and crushed mollusk shells in fixed-bed columns for the treatment of synthetic landfill leachate. J Mater Cycles Waste Manage.

[CR6] Costanzo SD, Watkinson AJ, Murby EJ, Kolpin DW, Sandstrom MW (2007). Is there a risk associated with the insect repellent DEET (N,N-diethyl-m-toluamide) commonly found in aquatic environments?. Sci Total Environ.

[CR7] Dos Santos FL, Campos B, de Araújo M, Goreti Rodrigues Vale M, Caramão EB (2004). Application of activated carbon in the characterization of nitrogen compounds and phthalates in a landfill leachate. Microchem J.

[CR8] European Commission (1976). European Directive 76/768/EEC and its successive amendments, basic act 31976L0768.

[CR9] Foo KY, Lee LK, Hameed BH (2013). Batch adsorption of semi-aerobic landfill leachate by granular activated carbon prepared by microwave heating. Chem Eng J.

[CR10] Gracia-Lor E, Martínez M, Sancho JV, Peñuela G, Hernández F (2012). Multi-class determination of personal care products and pharmaceuticals in environmental and wastewater samples by ultra-high performance liquid-chromatography-tandem mass spectrometry. Talanta.

[CR11] Harvey PW, Everett DJ (2004). Significance of the detection of esters of p-hydroxybenzoic acid(parabens) in human breast tumours. J Appl Toxicol.

[CR12] Inspection of Environmental Protection, Inspectorate of Environmental Protection in Bialystok (2013). State of the Environment Report Podlaskie Province in 2011–2012.

[CR13] Kalka J (2012). Landfill leachate toxicity removal in combined treatment with municipal wastewater. Sci World J.

[CR14] Kalmykova Y, Björklund K, Strömvall A-M, Blom L (2013). Partitioning of polycyclic aromatic hydrocarbons, alkylphenols, bisphenol A and phthalates in landfill leachates and stormwater. Water Res.

[CR15] Kotowska U, Kapelewska J, Sturgulewska J (2013). Determination of phenols and pharmaceuticals in municipal wastewaters from Polish treatment plants by ultrasound-assisted emulsification–microextraction followed by GC–MS. Environ Sci Pollut R.

[CR16] Kumar V, Nakada N, Yasojima M, Yamashita N, Johnson AC, Tanaka H (2009). Rapid determination of free and conjugated estrogen in different water matrices by liquid chromatography–tandem mass spectrometry. Chemosphere.

[CR17] Kurata Y, Ono Y, Ono Y (2008). Occurrence of phenols in leachates from municipal solid waste landfill sites in Japan. J Mater Cycles Waste Manage.

[CR18] Kwan CS, Takada H, Mizukawa K, Torii M, Koike T, Yamashita R, Rinawati Saha M, Santiago EC (2012). PBDEs in leachates from municipal solid waste dumping sites in tropical Asian countries: phase distribution and debromination. Environ Sci Pollut R.

[CR19] Liu Y-S, Ying G-G, Shareef A, Kookana RS (2011). Simultaneous determination of benzotriazoles and ultraviolet filters in ground water, effluent and biosolid samples using gas chromatography–tandem mass spectrometry. J Chromatogr A.

[CR20] Liu Y-S, Ying G-G, Shareef A, Kookana RS (2012). Occurrence and removal of benzotriazoles and ultraviolet filters in a municipal wastewater treatment plant. Environ Pollut.

[CR21] Loos R, Tavazzi S, Paracchini B, Canuti E, Weissteiner C (2013). Analysis of polar organic contaminants in surface water of the northern Adriatic Sea by solid-phase extraction followed by ultrahigh-pressure liquid chromatography–QTRAP® MS using a hybrid triple-quadrupole linear ion trap instrument. Anal and Bioanal Chem.

[CR22] Ma JJ, Du X, Zhang JW, Li JC, Wang LZ (2009). Ultrasound-assisted emulsification–microextraction combined with flame atomic absorption spectrometry for determination of trace cadmium in water samples. Talanta.

[CR23] Nomngongo PN, Catherine Ngila J, Msagati TAM, Gumbi BP, Iwuoha EI (2012). Determination of selected persistent organic pollutants in wastewater from landfill leachates, using an amperometric biosensor. Phys Chem Earth, Parts A/B/C.

[CR24] Odusanya DO, Okonkwo JO, Botha B (2009). Polybrominated diphenyl ethers (PBDEs) in leachates from selected landfill sites in South Africa. Waste Manage.

[CR25] Pietrogrande MC, Basaglia G (2007). GC-MS analytical methods for the determination of personal-care products in water matrices. TrAC Trend Anal Chem.

[CR26] Preiss A, Berger-Preiss E, Elend M, Gerling S, Kühn S, Schuchardt S (2012). A new analytical approach for the comprehensive characterization of polar xenobiotic organic compounds downgradient of old municipal solid waste (MSW) landfills. Anal Bioanal Chem.

[CR27] Regueiro J, Llompart M, Garcia-Jares C, Garcia-Monteagudo JC, Cela R (2008). Ultrasound-assisted emulsification–microextraction of emergent contaminants and pesticides in environmental waters. J Chromatogr A.

[CR28] Regueiro J, Becerril E, Garcia-Jares C, Llompart M (2009). Trace analysis of parabens, triclosan and related chlorophenols in water by headspace solid-phase microextraction with in situ derivatization and gas chromatography–tandem mass spectrometry. J Chromatogr A.

[CR29] Regueiro J, Llompart M, Psillakis E, Garcia-Monteagudo JC, Garcia-Jares C (2009). Ultrasound-assisted emulsification–microextraction of phenolic preservatives in water. Talanta.

[CR30] Renou S, Givaudan JG, Poulain S, Dirassouyan F, Moulin P (2008). Landfill leachate treatment: review and opportunity. J Hazard Mater.

[CR31] Rosi-Marshall EJ, Royer TV (2012). Pharmaceutical compounds and ecosystem function: an emerging research challenge for aquatic ecologists. Ecosystems.

[CR32] Streets S (2008) Endocrine Disrupting Compounds: A Report to the Minnesota Legislature. Minnesota Pollution Control Agency. http://www.pca.state.mn.us/index.php/view-document.html?gid=3943

[CR33] Trenholm RA, Vanderford BJ, Drewes JE, Snyder SA (2008). Determination of household chemicals using gas chromatography and liquid chromatography with tandem mass spectrometry. J Chromatogr A.

[CR34] Yamamoto T, Yasuhara A, Shiraishi H, Nakasugi O (2001). Bisphenol A in hazardous waste landfill leachates. Chemosphere.

[CR35] Zhang Y, Lee HK (2012). Determination of ultraviolet filters in water samples by vortex-assisted dispersive liquid-liquid microextraction followed by gas chromatography–mass spectrometry. J Chromatogr A.

[CR36] Zhang Y, Lee HK (2013). Determination of ultraviolet filters in environmental water samples by temperature-controlled ionic liquid dispersive liquid-phase microextraction. J Chromatogr A.

[CR37] Zhang C, Eganhouse RP, Pontolillo J, Cozzarelli IM, Wang Y (2012). Determination of nonylphenol isomers in landfill leachate and municipal wastewater using steam distillation extraction coupled with comprehensive two-dimensional gas chromatography/time-of-flight mass spectrometry. J Chromatogr A.

